# Novel insights into the molecular nature of neurofilament light polypeptide species in cerebrospinal fluid

**DOI:** 10.1093/braincomms/fcaf129

**Published:** 2025-04-02

**Authors:** Bruno Becker, Johan Gobom, Gunnar Brinkmalm, Ulf Andreasson, Francisco J Meda, Henrik Zetterberg, Kaj Blennow

**Affiliations:** Institute of Neuroscience and Physiology, Department of Psychiatry and Neurochemistry, The Sahlgrenska Academy at University of Gothenburg, S-43180 Mölndal, Sweden; Clinical Neurochemistry Laboratory, Sahlgrenska University Hospital, S-43180 Mölndal, Sweden; Institute of Neuroscience and Physiology, Department of Psychiatry and Neurochemistry, The Sahlgrenska Academy at University of Gothenburg, S-43180 Mölndal, Sweden; Clinical Neurochemistry Laboratory, Sahlgrenska University Hospital, S-43180 Mölndal, Sweden; Institute of Neuroscience and Physiology, Department of Psychiatry and Neurochemistry, The Sahlgrenska Academy at University of Gothenburg, S-43180 Mölndal, Sweden; Clinical Neurochemistry Laboratory, Sahlgrenska University Hospital, S-43180 Mölndal, Sweden; Institute of Neuroscience and Physiology, Department of Psychiatry and Neurochemistry, The Sahlgrenska Academy at University of Gothenburg, S-43180 Mölndal, Sweden; Clinical Neurochemistry Laboratory, Sahlgrenska University Hospital, S-43180 Mölndal, Sweden; Institute of Neuroscience and Physiology, Department of Psychiatry and Neurochemistry, The Sahlgrenska Academy at University of Gothenburg, S-43180 Mölndal, Sweden; Institute of Neuroscience and Physiology, Department of Psychiatry and Neurochemistry, The Sahlgrenska Academy at University of Gothenburg, S-43180 Mölndal, Sweden; Clinical Neurochemistry Laboratory, Sahlgrenska University Hospital, S-43180 Mölndal, Sweden; UCL Institute of Neurology, Department of Molecular Neuroscience, University College London, Queen Square, London WC1N 3BG, UK; UK Dementia Research Institute at UCL, London NW1 3BT, UK; Hong Kong Center for Neurodegenerative Diseases, Hong Kong Science Park, Shatin, N.T., Hong Kong, China; Wisconsin Alzheimer’s Disease Research Center, University of Wisconsin School of Medicine and Public Health, University of Wisconsin-Madison, Madison, WI 53792, USA; Institute of Neuroscience and Physiology, Department of Psychiatry and Neurochemistry, The Sahlgrenska Academy at University of Gothenburg, S-43180 Mölndal, Sweden; Clinical Neurochemistry Laboratory, Sahlgrenska University Hospital, S-43180 Mölndal, Sweden; Paris Brain Institute, ICM, Pitié-Salpêtrière Hospital, Sorbonne University, FR-75013 Paris, France; Neurodegenerative Disorder Research Center, Division of Life Sciences and Medicine, and Department of Neurology, Institute on Aging and Brain Disorders, University of Science and Technology of China and First Affiliated Hospital of USTC, Hefei, PR China

**Keywords:** neurofilament light, NFL, Uman MAb, aggregates, size exclusion chromatography

## Abstract

The quantification of neurofilament light polypeptide (NFL) in biofluids is being clinically used to detect and grade general neuronal damage in neurodegenerative diseases and quantify neuronal injury during acute events like traumatic brain injury. Specific assays that target only particular molecular breakdown products of neurofilaments have the potential to distinguish between various pathologies. Nevertheless, the molecular structure of neurofilament light polypeptide in cerebrospinal fluid remains to be elucidated. We characterized neurofilament light polypeptide in cerebrospinal fluid by size-exclusion chromatography, Western blotting and mass spectrometry. Neurofilament light polypeptide in cerebrospinal fluid was found to be composed of aggregates of fragments of the full-length molecule. These aggregates were sensitive to reduction by dithiothreitol and dissociated to monomeric fragments of 6–12 kDa (Western blot), covering most of the coiled-coil domains of neurofilament light polypeptide. Since only cysteine residues can form disulfide bonds, this points to a role of the single cysteine 322 for maintaining the stability of the aggregates. The sequence region covered by the identified fragments ended just a few amino acids C-terminally of the coiled-coil region at a site which had been previously mapped to a calpain cleavage site in the glial fibrillary acidic protein, which is highly homologous to neurofilament light polypeptide in the coiled-coil region. This cleavage site was also confirmed to be present in bovine neurofilament light polypeptide by *in vitro* digestion of purified neurofilament light polypeptide with calpain-1. The difference of the molecular weights of the reduced and non-reduced forms of neurofilament light polypeptide suggests that neurofilament light polypeptide in CSF consists of disulfide-linked aggregated fragments, most likely tetramers, or alternately dimers in a complex with another binding partner.

## Teaser

Using classical biochemical methods and mass spectroscopy, Becker *et al*. characterized aggregate forms of the neurofilament light protein in cerebrospinal fluid. Neurofilament light protein was found to be truncated, forming disulfide-linked complexes. Knowledge about these structures is of importance for the correct quantitation of neurofilament light protein in neurodegeneration biomarker assays.

## Graphical Abstract

**Graphical Abstract:**
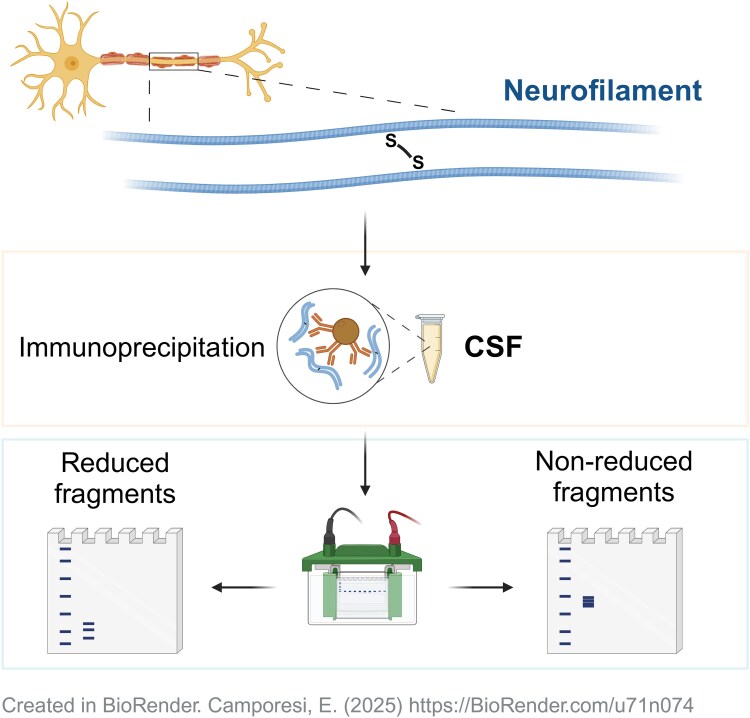

## Introduction

Neurofilaments are generally considered very stable components of the neuronal cytoskeleton, providing physical strength to the axons and a scaffold for organizing and structure the cell nucleus and organelles.^1-4^ Other functions, such as anchoring glutamatergic receptors in synaptic compartments and cross-linking to the other cytoskeletal filament systems are also being investigated.^3,5,6^ The neurofilaments belong to the class of intermediate filaments, with a thickness of about 10 nm, and are composed of heteropolymers of mainly three different neurofilament proteins, namely neurofilament light (NFL), neurofilament medium and neurofilament heavy. In the central nervous system, a fourth neurofilament protein, α-internexin^7^ is also found in assembled neurofilaments, and in the peripheral nervous system there is an additional neurofilament protein called peripherin.^7^ All variants of the neurofilament proteins share an overall similar structure, with an ordered central α-helical domain of high homology, bordered by disordered N-terminal domains and C-terminal domains of different lengths.

In mature neurofilaments, the different molecular chains of the neurofilament proteins are assembled to higher order structures to shape the filament structure. NFL can form parallel homodimers, or it can assemble with one of the other variants (e.g. neurofilament heavy or neurofilament medium) to a parallel heterodimer. These dimers then assemble in ‘anti-parallel’ orientation to form tetramers. Eight of those tetramers then form a cylindrical subunit, the unit length filament, which is considered the elementary unit of a neurofilament. Several such building blocks can then form neurofilaments by attaching to each other head to tail.^8^

In spite of the stability of neurofilaments, disassembled neurofilament proteins can be detected in biofluids, such as cerebrospinal fluid (CSF) and blood.^9-14^ In the case of NFL, only fragments but not the full-length protein have been detected. These were most likely produced by action of proteases such as calpain.^15^ Increased levels are often found in patients with neurodegenerative diseases and traumatic brain injury and NFL also increases with age. High levels are found in patients with Creutzfeldt–Jakob disease, amyotrophic lateral sclerosis, frontotemporal dementia, immune-mediated neuropathies (Guillain−Barré syndrome, multiple sclerosis),^16^ and typically, levels in Alzheimer’s disease patients^16,17^ are also increased, but to a lesser degree.

NFL has emerged as an important biomarker for neurodegeneration in samples from CSF and blood (plasma, serum). The currently available assays are based on immunodetection of the central helical region of NFL and are enabled by various technical platforms [Enzyme Linked Immunosorbent Assay (ELISA), Simoa, Lumipulse, ELLA, etc.].^11,13^ Even after years of using the NFL sandwich assays in clinical routine, it is still unclear which molecular species these assays are detecting in CSF or plasma. The concentrations determined by the various assay platforms correlate well but may differ in their absolute values as different calibration standards and different antibodies are being used. The high degree of correlation suggests that these platforms target very similar molecular species. This seems also likely because the epitopes of the antibodies in those sandwich assays have been tentatively mapped to coil 2 of the molecule.^18^

Both from a basic science and a diagnostic application viewpoint it is of interest to know which structures of intermediate filaments can be found in biofluids. A recent paper^19^ described a map of fragments of NFL found in CSF. Using immunoprecipitation and mass spectrometry, fragments from the α-helical core domain (coils 1A, 1B and 2B) and from the C-terminal of NFL were identified and further characterized. It remained untested whether these fragments appeared as monomeric molecules or as part of higher-order structures (unit length filaments, tetramers, dimers), which may have remained intact after the breakdown of neurofilaments during neurodegeneration.

Here, we investigate which molecular forms of NFL can be detected in pools of CSF with help of monoclonal antibodies^18^ targeting a similar region of NFL as the Uman antibodies of the commonly used ‘NF-light^™^’ ELISA CSF assay.^20^ By using a combination of size exclusion chromatography (SEC), immunoprecipitation, Western blot techniques and mass spectrometry (MS), we found that the NFL molecules targeted by these antibodies are most likely either tetramers of NFL fragments or dimers of NFL fragments in a complex with as of yet unknown binding partners.

## Materials and methods

### Antibodies

MAbs NFL21 and NFL23 were prepared in-house according to standard protocols and used in sandwich ELISA assays^21^ to quantitate NFL in CSF samples. MAbs 6H63 and 1B11 were obtained from EnCor Biotechnology, USA (cat. nr. MCA-6H63 and MCA-1B11, respectively). 6H63 was raised against recombinant immunogen representing part or all of coil2 region of NFL (details proprietary to EnCor Technology), whereas 1B11 was obtained using a purified NFL preparation from pig spinal cord.

### CSF samples

CSF samples were obtained as anonymized clinical samples from the neurochemistry laboratory at Sahlgrenska University hospital, Mölndal, Sweden. They were used either as individual samples or as pools.

For preparation of pools, CSF samples were categorized according to their NFL content into high NFL (>10 ng/mL) or low NFL (∼0.3–0.6 ng/mL) and the corresponding samples mixed to give pools of high and low NFL. After mixing, the pools were centrifuged at 4°C at 3000*×g* for 15 min. The supernatants were collected, avoiding the last mL, and samples were taken for determination of the NFL concentration. The pools were stored frozen at −20°C.

### Size exclusion chromatography of CSF

A Superdex Increase 200 (10 mm × 300 mm) column was equilibrated in 50 mM ammonium bicarbonate (freshly prepared, pH unadjusted) and calibrated by injection of SEC size markers (Sigma; #MWGF70) diluted in 50 mM ammonium bicarbonate. Flow 0.4 mL/min, in the cold room (∼6°C). For separation of CSF, a sample of 1 mL CSF (NFL conc. = 13.4 ng/mL by ELISA) was injected and 1-mL fractions collected. Analysis of NFL in fractions was done by our in-house NFL ELISA assay.^21^ The injection volume of CSF and of the calibrators were always kept the same to avoid miscalibration. For more accurate size estimation, a lower injection volume of CSF (0.5 mL), and an additional size marker at 669 kDa were used in an additional SEC separation of CSF. The size markers used were thyroglobulin, bovine (669 kDa; Sigma-Aldrich; #T9145), albumin, bovine (66 kDa; Sigma-Aldrich; #A8531), carbonic anhydrase, bovine (29 kDa; Sigma-Aldrich; #C7025), cytochrome C, horse (12.4 kDa; Sigma-Aldrich; #C7150) and aprotinin, bovine (6.5 kDa; Sigma-Aldrich; #A3886).

### Ultrafiltration of CSF

CSF samples (500 µL) of varying NFL content were concentrated in 3 K Ultraspin filters (Merck Millipore; #UFC500396) by spinning at 7500 × *g* at 4°C for 1 h (5- to 6-fold increase in concentration). The retentates were then analyzed for NFL via western blots.

### Reducing and non-reducing SDS-PAGE

For sample preparation, the samples were mixed with sample buffer (‘XT’, 4X; Bio-Rad; #1610791) and 1 M dithiothreitol (DTT, 20X; for reduced samples) or with an equivalent volume of water (for non-reduced samples). After heating to 70°C for 10 min., the samples were loaded on Criterion XT Bis-Tris gels (Bio-Rad) and electrophoresed in MES buffer. Prestained Sodium dodecyl-sulfate polyacrylamide gel electrophoresis (SDS-PAGE) molecular weight (MW) markers were used (SeeBlue Plus2, Life Technologies; #LC5925).

### Western blot procedure

SDS PAGE gels were first equilibrated for 20 min with light shaking in transfer buffer (NuPAGE^™^; Thermo Fisher Scientific; #NP0006-1) containing 20% (v/v) methanol). The electrophoretic transfer to a 0.2 µm nitrocellulose membrane (Amersham Protran; #10600001) was performed for 1 h in a semi-dry blotter unit (Scie-Plas, UK; #V20-SDB). Two layers of extra thick filter paper (Thermo Fisher Scientific; #88620) on each electrode, presoaked in transfer buffer, were used to sandwich the membrane and gel assembly between the electrodes. The transfer was carried out at 0.8 mA/cm^2^ constant current for 1 h at room temperature (RT). Blotted membranes were blocked for 1 h at RT either in 3% BSA (bovine serum albumin (Sigma-Aldrich; #A7906)) in PBST (phosphate-buffered saline with 0.05% Tween20 (Bio-Rad #1610781)) or in 5% low fat milk (BioRad; #170-6404) in PBST. After two quick rinses with PBST, the membranes were incubated overnight on a rocker at 4°C in primary antibody solution (0.5–1 µg/mL MAb in PBST with 0.1% BSA). After 3 washes with PBST (10 min each), the membranes were incubated for 1 hour at RT in secondary antibody solution, e.g. anti mouse IgG-HRP (Cell Signaling; #7076; dilution 1:8000 in PBST, 0.1% BSA). Alternatively, if the primary antibody was biotinylated, Enhanced Streptavidin-HRP (KemEnTech; #4740N) was used at a dilution of 1:20 000 in PBST, 0.1%BSA instead of the secondary antibody solution. After three washes in PBST (10 min each), the membranes were rinsed twice in water and placed with the blot side up on parafilm. Substrate mix ECL Select (GE; #RPN2235; for high sensitivity) or Clarity Western ECL Substrate (Bio-Rad; #1705061SP; for low background) was added for 2–5 min, before the membrane was placed wet between two clear plastic sheets for imaging on an imager LAS-3000 (Fujifilm).

### Immunoprecipitation

Aliquots (200 μL) of anti-mouse IgG magnetic beads (M-280 Dynabeads, Thermo Fisher Scientific; #11202D) were separately coated with monoclonal antibodies (MAbs) 1B11 and 6H63 (Encor Biotechnology, USA) at a ratio of 6 μg MAb per 200 μL bead suspension. The coated beads were washed 3 times with PBS, 0.05% Tween 20 (5 min/wash) and resuspended in 200 μL PBS, 0.05% Tween 20. The washed coated beads were then combined and used for immunoprecipitation. To 40 mL CSF pool with high NFL concentration (16.6 ng/mL by ELISA) 200 μL 10% Tween 20 and 400 μL of the coated bead mix were added. The tube with the immunoprecipitation mix was then rotated overnight at 4°C. After separation on a magnet, most of the supernatant, down to 4 mL, was discarded and the beads resuspended in those 4 mL. The bead suspension was then divided into 4 aliquots of 1 mL and then washed in a magnetic bead separator/washer (Kingfisher, Thermo Electron). Each bead aliquot was washed consecutively in 1 mL PBS with 0.05% Tween 20, 1 mL PBS, and 1 mL of 50 mM ammonium bicarbonate. Finally, the magnetically separated beads were incubated 10 min in 0.5 mL 0.5% formic acid to elute bound proteins and peptides. The 4 eluates were combined and aliquoted into 8 aliquots for evaporation on a SpeedVac concentrator (Savant SC210A, Thermo Scientific). Two of these aliquots were then used for MS analysis after digestion by Lys-C in reduced and non-reduced conditions, two more aliquots were analyzed by MS after separation in an SDS-PAGE gel and in-gel digestion (see below).

### Lys-C digestion (in solution)

One of the aliquots from the immunoprecipitation (MAbs 1B11 and 6H63) from high NFL CSF was used directly for digestion, the other one was first reduced with DTT and carbamidomethylated (with iodoacetamide) according to standard procedures for MS sample preparation. Digestion was performed in a volume of 50 µL using 1.5 µL of Lys-C (200 ng/µL; Promega, # VA1170) over night at 37°C. Finally, the digestion was stopped by addition of formic acid to 1%.

### Lys-C digestion (in gel)

Two aliquots of the immunoprecipitate (see above) were separated on a SDS-PAGE gel (one under reducing and one at non-reducing conditions). The two gel lanes were then cut each into 11 pieces representing the whole MW range from 200 kDa to < 3 kDa. Each piece of the reduced sample lane underwent separately reduction and carbamidomethylation (with 10 mM DTT and 55 mM iodoacetamide, respectively), followed by digestion with 375 ng Lys-C (Promega; #VA117A) at 37°C over night. The gel pieces of the non-reduced sample lane were processed the same way, but under non-reducing conditions, whereby DTT and iodoacetamide were replaced with buffer (25 mM ammonium bicarbonate). A detailed description of the procedure can be found in Supplementary Materials.

### Mass spectrometric analysis

LC-MS analyses were performed using an Ultimate 3000 RSLC-nano HPLC coupled to an Orbitrap Lumos mass spectrometer equipped with a high-field asymmetric waveform ion mobility spectrometry Pro interface via an EASY Spray electrospray ion source (all from Thermo Scientific). The spray voltage was 1900V. The HPLC was operated in trap column configuration, using a 5 mm × 300 µm PepMap C18 trap cartridge and a 500 mm × 75 µm EASY Spray C18 separation column (Thermo Scientific). Samples were loaded for 5 min in 0.05% TFA at a flow of 50 µL/min and separation was performed using a 60 min linear gradient of 4–35% Buffer A (0.1% formic acid) and Buffer B (80% acetonitrile, 0.1% formic acid) at a flow of 300 nL/min. The trap cartridge was kept at 30°C, and the separation column was operated at 50°C. The field asymmetric waveform ion mobility spectrometry was operated in the standard resolution mode. To improve sequence coverage, duplicate analysis runs were performed of each sample, using four different compensation voltages in each run (40, 50, 60 and 70 V or 45, 55, 65 and 75 V). The mass spectrometer was operated in the positive ion mode, recording Orbitrap MS1 spectra at 120 k resolution and data dependent Orbitrap MS2 spectra at 30 k resolution (1.6 Th quadrupole isolation window, 30% normalized HCD collisional energy, 100% AGC target, 1.5 s cycle time).

Protein/peptide identification was performed using PEAKS 11 (Bioinformatics Solutions Inc.). The human or bovine subsets of the SwissProt database was searched using the following settings: Parent Mass Error Tolerance: 10 ppm; Fragment Mass Error Tolerance: 0.05 Da; Enzyme: Lys-C; Max Missed Cleavages: 2; semi-specific cleavage. Carbamidomethylation and carbamidomethylated DTT adduct of cysteine residues were set as variable modifications. Validation of peptide identification was performed using the target-decoy method, and reported peptides were identified at 1% FDR on the PSM level.

### NFL ELISA

Nunc Maxisorp plates (frames with inserts No 469914, Thermo Sci) were coated in 50 mM sodium bicarbonate buffer, pH 9.6 with 0.5 μg/mL NFL21 monoclonal antibody^21^ overnight at 4°C. After washing with PBS, 0.05% Tween 20, the plates were blocked with 1% BSA (Sigma-Aldrich; #A4503) for 1 h. Binding of samples was done overnight at 4°C. Biotinylated detection antibody was either monoclonal antibody NFL23 (0.4 μg/mL in PBS, 0.1% BSA; in-house) or NFL21 (in-house; for homogeneous assay format). Signal generation was via streptavidin-coupled horseradish peroxidase (SA-HRP) and 3,3′,5,5′-tetramethylbenzidine. Signals were compared against those obtained from dilutions of purified bovine NFL protein.

## Results

### NFL in CSF pools from patients is of higher order structure

**Figure 1:**
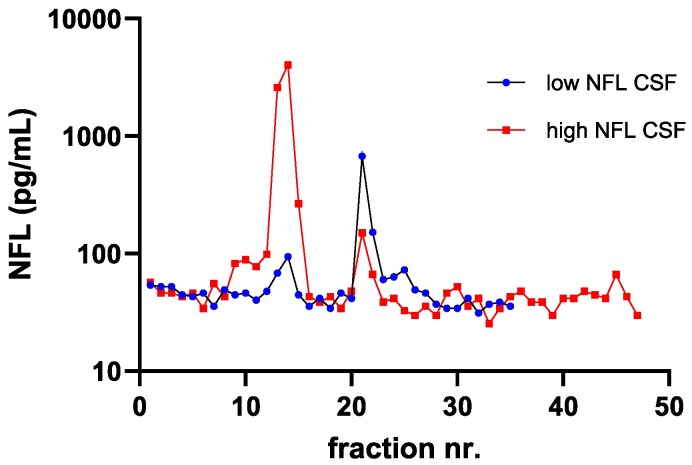
**Analysis of size-exclusion chromatography fractions** (1 mL) of CSF pools of high (c = 16 600 pg/mL; red) and low (c = 230 pg/mL; blue) concentrations of NFL by sandwich ELISA ‘NFL21_NFL23’ (using MAb NFL21 for capture and MAb NFL23 for detection, respectively). Lower limit of quantitation: ∼50 pg/mL. Single samples (*n* = 1) were analyzed.

We initially used SEC to determine the molecular size of NFL in CSF. Figure 1 shows the analysis of fractions obtained after SEC of two CSF samples, each consisting of pools of individual samples with high and low concentrations of NFL, respectively, by our in-house sandwich NFL ELISA^21^ (NFL21 MAb for capture and NFL23 for detection).

Almost all of the NFL ELISA signal from the high NFL pool was detected in fractions 13–14 corresponding to a MW of approx. 45 kDa. Additionally, weaker signals were detected in earlier fractions 9–10 and around later fractions 21–22 (red graph in Fig. 1). The later fractions corresponded to a MW of around 8 kDa, whereas the MW of the earlier fractions was difficult to estimate due to non-linearity of the log MW versus elution volume calibration curve at the high range of MW (data not shown). The peak heights of the smaller peaks (fractions 21–22) were only about 1–2% of the main peak. The low-NFL sample was analyzed to serve as a control (blue graph in Fig. 1). Indeed, the main peak at fractions 13–14 was now only about 2% of the peak height of the corresponding peak of the high-NFL sample, as was expected for a low NFL control sample. Interestingly, the low-NFL sample showed a larger peak in fraction 21 (corresponding to ∼8 kDa).

**Figure 2:**
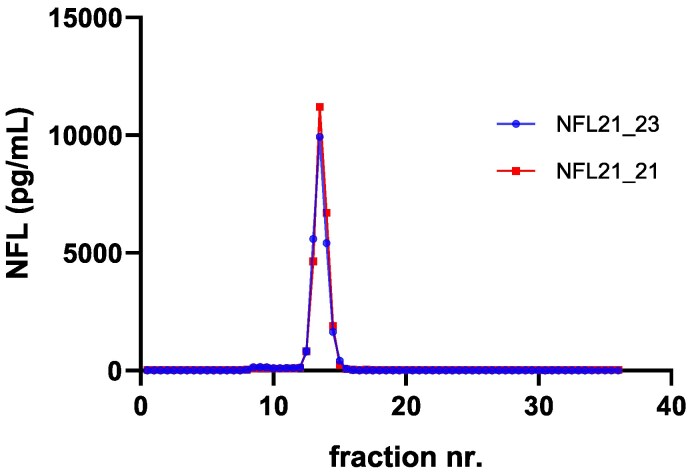
**Size exclusion chromatography profile of NFL in pooled CSF.** Fractions were analyzed by the in-house NFL sandwich ELISA assay ‘NFL21_NFL23’ (using MAb NFL21 for capture and MAb NFL23 for detection, respectively; see blue line) as well as the homogeneous assay ‘NFL21_NFL21’ (using mAb NFL21 both for capture and detection; see red line). NFL species detected were eluting at ∼12.5–14.5 mL (main peak) and in earlier fractions at 8.5–9.5 mL (small peak). Single samples (*n* = 1) were analyzed.

Previous work has shown that higher order intermediate structures of NFL exist in CSF, as they can be detected by a homogeneous ELISA.^17^ We confirmed this by analyzing size-separated fractions of the high-NFL sample by our in-house ELISA for NFL (NFL21/NFL23; blue line in Fig. 2) and by its homogeneous variant (ELISA NFL21/NFL21; red line in Fig. 2). As can be seen in Fig. 2, the signals for both ELISAs almost completely overlapped, confirming that NFL in the CSF pool is of higher order structure (i.e. molecules of NFL in CSF are dimeric or multimeric) since the homogeneous assay can only detect species that have at least two binding sites for the same antibody.^17^

### Western blot detection of NFL fragments in individual CSF samples

**Figure 3:**
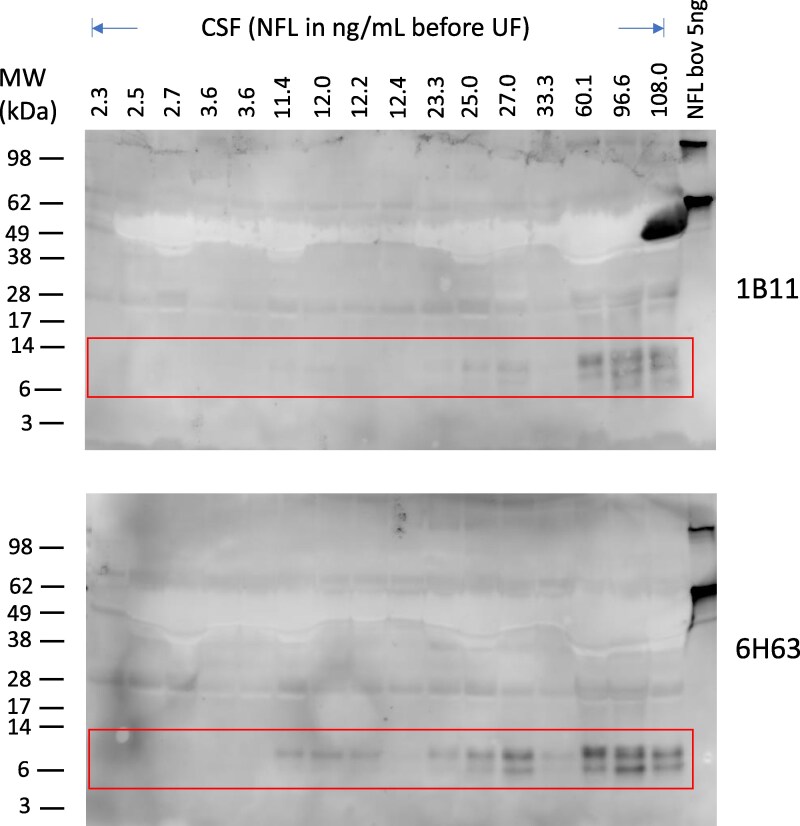
**Western blot of concentrated reduced individual patient CSF** samples ranked by increasing NFL concentrations (ng/mL as determined by ELISA). The MAb 1B11 detected at least three bands, whereas MAb 6H63 detected two bands in the size region of approx. 6–12 kDa (see boxed region). UF, ultrafiltration. See also Supplementary Fig. 3 for an uncropped image of this Western blot. Molecular weights of SDS-PAGE size markers are indicated in kDa.

Attempts to detect NFL-specific bands on Western blots of CSF were initially unsuccessful due to the low concentrations of NFL present and the high background of other antibody binding proteins. Only after preconcentration of CSF by ultrafiltration via spin filters and the use of the MAbs 1B11 and 6H63, we could detect bands on Western blots (Fig. 3) with intensities that correlated with the NFL content in individual patient samples, as measured by ELISA. The MAbs 1B11 and 6H63 have epitopes in the α-helical domain 2 of NFL, very similar to the widely used NFL antibodies of the Uman ELISA assay for NFL^18^ or our in house NFL21_23 assay.^21^ As shown in Fig. 3 (boxed regions), at least three specific bands were detected by MAb 1B11 and at least two bands by MAb 6H63, all in the size range of approx. 6–12 kDa. The intensity of the bands correlated roughly with the NFL concentration in the samples, with two exceptions: two samples (of concentration 12.4 ng/mL and 33.3 ng/mL before ultrafiltration) did not or only weakly show such bands, although preceeding adjacent lanes of lower concentrations of NFL did show bands. The reason for this discrepancy between Western blot and ELISA result is unknown, but it could indicate that the Western blot can potentially reveal patterns of NFL fragments which the ELISA assay (which uses different MAbs) might miss.

### Immunoprecipitation for NFL yields the same pattern of bands as direct western blot of CSF

**Figure 4:**
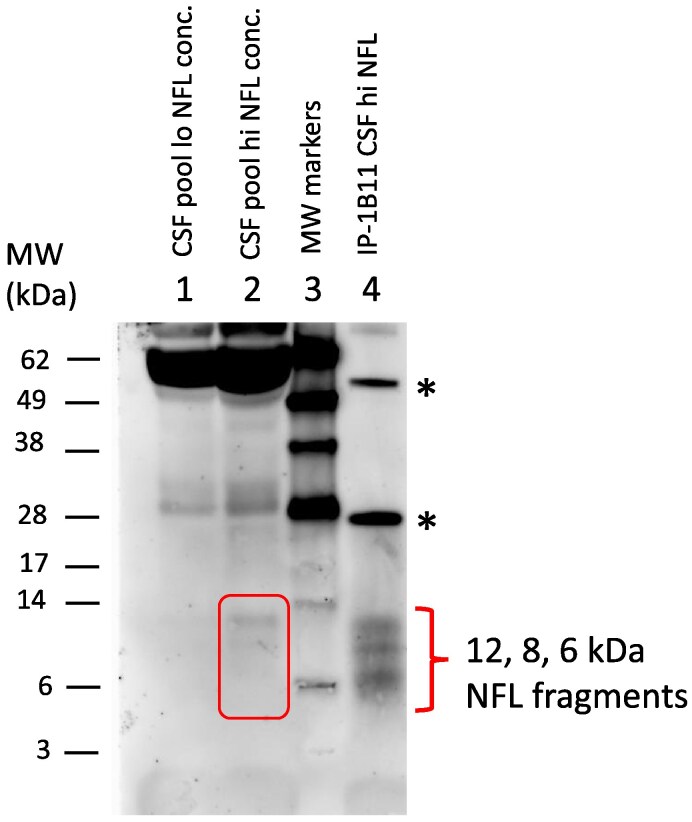
**Western blot of reduced samples of concentrated CSF** (lanes 1, 2) using MAb 1B11. Lane 1 contained concentrated CSF from a pool of low-NFL samples, whereas lane 2 contained concentrated CSF from a pool of high NFL. Lane 3, size markers. Lane 4, CSF pool (different pool compared to that used for lane 1 and 2) immunoprecipitated by MAb 1B11. Note that the bands detected in the IP sample appeared to be of the same MW as those in the concentrated CSF pool sample of lane 2. *, bands most likely representing heavy- and light-chain IgGs from antibodies used for immunoprecipitation. IP, immunoprecipitate. See also Supplementary Fig. 4 for an uncropped image of this Western blot. Molecular weights of SDS-PAGE size markers are indicated in kDa.

Direct Western blotting of CSF is often problematic due to the background of other proteins present. To reduce that background, we performed an immunoprecipitation experiment prior to the Western blot, using MAb 1B11 for immunoprecipitation and compared on a Western blot (Fig. 4) using 1B11 the band pattern obtained from the eluate from the immunoprecipitation to the pattern obtained without prior immunoprecipitation (‘direct’ Western blot). Three bands in the expected size range (Fig. 4, lanes 2 and 4; 6–12 kDa), were obtained, very similar but with higher intensity and less background than the band pattern shown in Fig. 3 in which CSF was directly blotted (panel 1B11).

### The apparent size of NFL in CSF depends on the redox state of NFL

**Figure 5:**
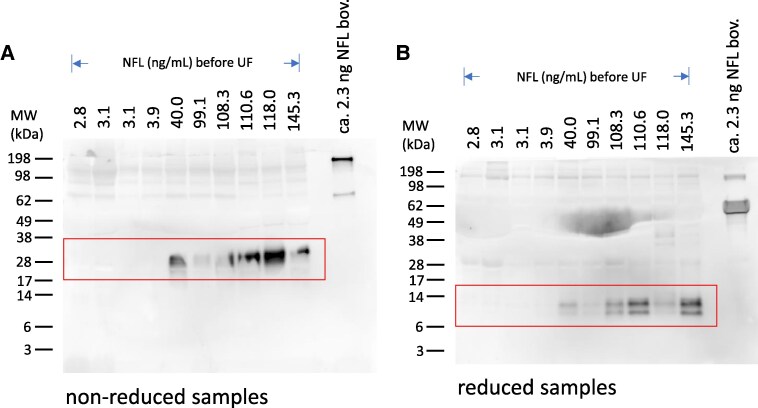
**The MW of NFL species appears 3–4 × higher in non-reduced versus reduced samples.** Western blots of individual patient CSF samples ranked by increasing NFL concentrations (ng/mL as determined by ELISA). Detection using MAb 6H63 shows the presence of NFL species at apparently ∼25–35 kDa [non-reduced samples; (**A**)] and at ∼8–12 kDa [reduced samples; (**B**)]. See also Supplementary Fig. 5 for an uncropped image of this Western blot. Molecular weights of SDS-PAGE size markers are indicated in kDa. UF, ultrafiltration.

NFL contains one cysteine in its sequence, at position 322 in domain 2B of the molecule, possibly involved in dimerization. We therefore analyzed a set of spin filter-concentrated individual CSF samples on Western blots (Fig. 5) using MAb 6H63 also at non-reducing conditions, which should leave a disulfide bridge between two molecules intact (Fig. 5A). We compared the results to those obtained from the same samples with prior reduction (Fig. 5B).

As expected from one of the previous Western blots (Fig. 3, blot ‘6H63’), several bands of apparent sizes in the range of ∼8–12 kDa were again detected in the blot containing reduced samples (Fig. 5B). In contrast, the non-reduced samples did not show such bands but instead bands at an about 3–4-fold higher MW of about 25–35 kDa (Fig. 5A, boxed area). Again, not all patient samples showed fragment bands of expected intensity, as previously also seen in Fig. 3. For example, the sample with 118.0 ng/mL NFL showed a strong band as a non-reduced sample, but only weak bands after reduction.

### The Uman diagnostics MAb UD3 detects only non-reduced species of NFL

**Figure 6:**
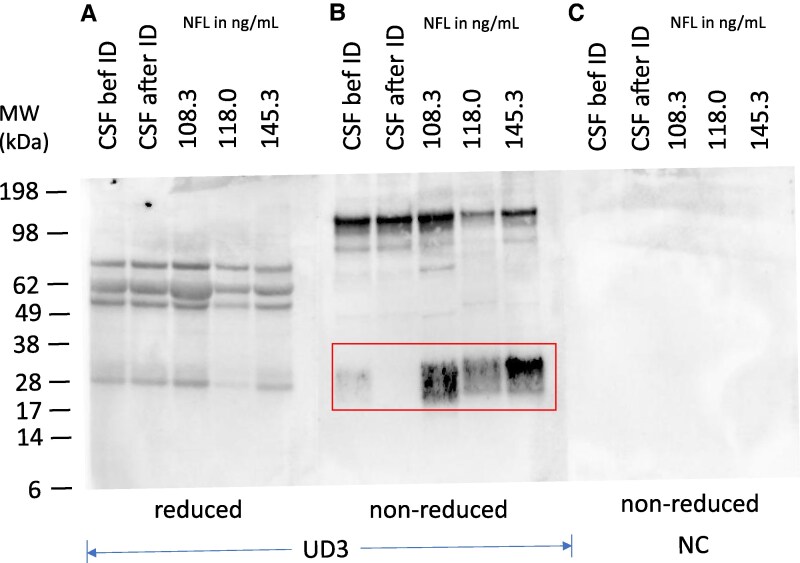
**Uman MAb UD3 detects non-reduced forms of NFL but not the reduced forms.** CSF samples were separated on SDS-PAGE and blotted after reduction (**A**, ‘reduced’) and as non-reduced samples (**B**. ‘non-reduced’). The CSF samples were: CSF pool (‘CSF before ID’ and ‘CSF after ID’) and individual CSF samples with the NFL content (ng/mL) as indicated. NFL concentration of the CSF pool was 16.6 ng/mL. For the immunodepletion, a 1:1 mixture of magnetic beads coated with either MAbs 1B11 or 6H63 was used. (**C**) NC, negative control (primary antibody omitted); Boxed region, NFL species detected at non-reducing conditions. ID, Immunodepletion. See also Supplementary Fig. 6 for an uncropped image of this Western blot. Molecular weights of SDS-PAGE size markers are indicated in kDa.

The currently most widely used immunoassay to detect NFL in CSF or plasma is based on MAbs originally described by Norgren, *et al.*^22,23^ MAb 47:3 is in this assay used as a capture antibody, whereas MAb 2:1 is used as the detector antibody. The latter is also commercially available from Quanterix as ‘UD3’, a biotinylated preparation of MAb 2:1. We used MAb UD3 to further confirm specificity of the previously seen bands of Fig. 5 by Western blotting. However, detection of specific bands by UD3 was dependent on the redox state of the CSF samples blotted. When reduced samples of CSF were analyzed using UD3, only non-specific bands were obtained (Fig. 6A). They were deemed non-specific because nearly the same band pattern was obtained across samples of varying NFL content and the major rounded band at 62 kDa most likely represented serum albumin. But when non-reduced CSF samples were analyzed (Fig. 6B, lanes 3–5), somewhat fuzzy bands around 30 kDa of similar sizes as in Fig. 5 (which used MAb 6H63 for detection) were observed. To show that NFL fragments detected by UD3 indeed also carried the epitopes for the Encor MAbs 1B11 and/or 6H63, we compared on the same Western blot (Fig. 6, lanes ‘CSF bef ID’ and ‘CSF after ID’) the fragment pattern of a pool of CSF before and after immunodepletion (ID) by a mix of 1B11 and 6H63—see Fig. 6B. It is clear that the mixture of the Encor Mabs 1B11 and 6H63 could immunodeplete NFL, so that the NFL fragments seen in Fig. 6B, lane ‘CSF bef ID’ were no longer detectable on the Western blot by UD3 after immunodepletion (Fig. 6B, lane ‘CSF after ID’).

### Identification of NFL fragments by IP-MS

**Figure 7:**
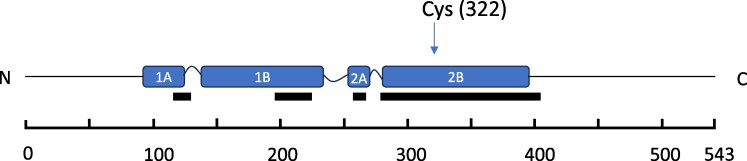
**Sequence coverage of NFL by peptides identified in lys-C digests of immunoprecipitates from CSF.** Aliquots of the immunoprecipitate were either digested directly or after reduction and carbamidomethylation by iodoacetamide. Domain locations (in blue) from Uniprot entry P07196, and regions covered by all identified peptides from both non-reduced and reduced/carbamidomethylated immunoprecipitates (black boxes underneath domain locations). At the bottom the amino acid numbers are indicated by a scale. The position of the single cysteine at aa 322 is indicated by an arrow. N, amino-terminal end; C, C-terminal end of sequence; 1A, 1B, 2A, 2B denote α-helical domains of NFL. See Supplementary Table 1, Supplementary Fig. 1 and Supplementary Fig. 2, for details on sequence coverage.

To further characterize the NFL fragments present in a CSF pool, we performed immunoprecipitation using a mixture of the Encor Biotechnology MAbs 1B11 and 6H63 and analyzed after digestion by Lys-C the eluate by mass spectrometry, as shown in Fig. 7 and in more detail in Supplementary Materials (Supplementary Table 1 and Supplementary Figs 1 and 2).

Overall, the peptides covered parts of coil 1A, 1B, 2A and the complete coil 2B. Peptides representing the linker regions between the coils as well as the N-terminal part and most of the C-terminal tail were not found. The most N-terminal peptide identified starts at position 117 (in coil 1A). This is followed by two peptides in coil 1B (aa 198–211; 212–224) and one peptide in coil 2A (aa 260–267). Coil 2B and a few amino acids of the beginning of the C-terminal tail are completely represented by a series of peptides in the range of aa 282–406 (coil 2B begins at aa 281 and ends at aa 396). Interestingly, peptides of neurofilament heavy, neurofilament medium or α-internexin were not detected indicating that only homopolymers of NFL were precipitated by those MAbs or that heteropolymers with other NFL subtypes were not present in the CSF sample. This analysis was performed using an aliquot of the immunoprecipitated CSF pool that had been digested directly with Lys-C; another aliquot was first reduced with DTT and then carbamidomethylated with iodoacetamide before digestion with Lys-C. This more restrictive enzyme was preferred to trypsin for digestion to yield larger cleavage products which are more likely to be retained by the liquid chromatography column. The peptide coverage for NFL is shown in Fig. 7 and in Supplementary Materials, Supplementary Table 1. In the reduced and carbamidomethylated sample, five Cys-containing peptides were identified, while in the non-reduced sample, only a single Cys-containing peptide was identified, and with much lower peak area (Supplementary Materials, Supplementary Fig. 2). Among the peptides found in the reduced and carbamidomethylated sample, there was a carbamidomethylated DTT adduct of the cysteine-peptide which was even more abundant than the ordinary carbamidomethylated cysteine peptide (without a DTT adduct). Since DTT selectively reacts with disulfides (but not free thiols), this suggests that the cysteine containing fragment had been linked in the CSF sample to another peptide via a disulfide bridge. It was however not possible to identify the binding partner of NFL that was linked via the -S-S- bridge.

**Figure 8:**
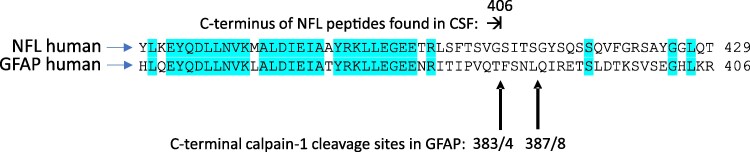
**Partial sequence alignment of human NFL with human GFAP with *in vitro* identified calpain-1 cleavage sites indicated.** Identical amino acid residues are highlighted in blue. Sequences and alignment are from Uniprot. GFAP, glial fibrillary acidic protein. For details on peptides identified by *in vitro* digestion of bovine NFL, see Supplementary Table 2.

No peptides were found covering the C-terminal sequence beyond amino acid (aa) 406, which suggests that a major cleavage site may be located here. Indeed, a sequence alignment of human NFL to human GFAP shows that this C-terminal end of human NFL at aa 406 corresponds to one of the two major C-terminal human GFAP cleavage sites by calpain (see alignment in Fig. 8) as reported in Yang *et al*.^24^

Indeed, we could confirm by mass spectrometry the presence of an analogous calpain-1 cleavage site by *in vitro* digestion of purified bovine NFL with calpain-1, followed by a Lys-C digestion (see Supplemental Materials, Supplementary Table 2). A fairly prominent peptide, ^393^LLEGEETRLSFTSVG^407^, was identified. The amino acid position 407 of bovine NFL corresponds to aa 406 of human NFL due to a one amino acid insertion after position 250 in the bovine NFL sequence. Bovine and human NFL are highly homologous, which makes it reasonable to expect that the *in vitro* cleavage results are transferable to human NFL as well.

We also confirmed the presence of NFL peptides by mass spectrometry in SDS-PAGE gel lanes in the size range of the NFL bands seen on Western blots at non-reducing conditions. The gel lane with the separated NFL immunoprecipitate was cut into 11 segments representing the full molecular weight range of separated proteins and each gel segment was digested with Lys-C, and the resulting peptides extracted and then analyzed by MS. NFL was identified in the gel band corresponding to MW 17–26 kDa (identified peptides: ^332^QLQELEDK^339^, ^371^EYQDLLNVK^379^, ^380^MALDIEIAAYRK^391^). A control Western blot of the immunoprecipitate confirmed the presence of NFL fragments in just the same MW range (data not shown). No NFL peptide was found outside this gel region. Thus, the MW of the NFL species identified in this immunoprecipitated CSF sample was narrowed down to 17–26 kDa and confirmed the identity of the NFL bands in the non-reduced Western blots, which migrated in the range of ∼25–35 kDa (as in Fig. 5A). However, no conclusive mass spectrometry results were obtained due to technical reasons from an analogous in-gel digestion experiment when another aliquot of the NFL immunoprecipitate was reduced prior to the separation on SDS-PAGE.

## Discussion

The assembly of neurofilaments has been described as a stepwise process, involving first the formation of NFL dimers from monomers, then their alignment to tetramers,^25^ eight of which then form unit-length cylindrical assemblies.^2^ The latter ones are considered the building blocks of neurofilaments. During the breakdown of the filaments in neurodegenerative or other conditions (e.g. aging and traumatic brain injury), each of these intermediates or some of their breakdown products may be released from the fibril and eventually be detectable in CSF or blood. Alternatively, but perhaps less likely, monomeric NFL (or fragments thereof) could be released to the interstitial fluid and then realign to dimers, tetramers and higher order structures. The mechanism of initial neurofilament breakdown is still unclear. Filament severing proteins have not been identified, and the breakdown could differ in physiological (aging) processes and in various diseases. The balance of phosphorylation and dephosphorylation of intermediate filament protein in its N-terminal segment may lead to filament disassembly.^26^ Alternatively, proteolysis, e.g. by calpain, cathepsins or other enzymes may also lead to instability of the filaments and consequently result in the release of intermediate filament substructures. A previous report mapped neurofilament fragments that can be found in CSF by mass spectrometric detection of immunoprecipitated neurofilament light peptides.^19^ Various tryptic/Lys-C fragments were found along the α-helical core region and at the very C-terminal end.

When we performed an immunoprecipitation of a CSF pool, using two MAbs from Encor Biotechnology (1B11 and 6H63), we found several peptides in the Lys-C digested eluate that covered the domain 2B completely as well as parts of domains 1A, 1B and 2A. The sequence coverage was somewhat complementary to that found by^19^ as we found most of the peptides in region 2B, whereas they found most of the peptides corresponding to region 1A, 1B and only some peptides to 2B as well as to the very C-terminal end. We presume that the difference is due to the different MAbs and/or the different CSF pools that have been used. In both cases, no N-terminal peptides were found, most likely because N-terminal capture antibodies were not available for immunoprecipitation, or such fragments may not exist at a detectable concentration in CSF.

Knowing that we can detect most of the sequence of the coiled core region of NFL in CSF raises new questions regarding the significance of the lack of representation of the linker regions and most of the N- and C- terminal domains. Are these regions much more susceptible to enzymatic cleavage or were the corresponding peptides merely undetectable because of technical limitations? Also, which size of fragments are present in CSF and do they have any quaternary structures? To answer some of these questions, we report here the detection of multimeric assemblies of neurofilament fragments in CSF, most likely tetramers, as the apparent size of the fragments shifts roughly 4-fold between non-reducing and reducing conditions in SDS-PAGE. Interestingly, the apparent tetramer structures remain intact even under the denaturing conditions of SDS-PAGE. The shift of apparent molecular weight by reduction indicates that a disulfide bridge via the single cysteine at position 322 is essential for stability of the complex under denaturing conditions. The approximately 4-fold shift indicates that the tetramer would consist of a non-covalent arrangement of two disulfide bonded NFL fragments or a NFL fragment bound via a disulfide bond to a non-NFL molecule. NFL dimers have been described as parallel dimers of α-helical coils which are coiled around each other and held together by strings of opposing patches of hydrophobic amino acid residues.^8^ This structural motif is characteristic of α-helical coiled-coils, and it appears when hydrophobic amino acids occupy in a periodic pattern the first and forth amino acid positions in a series of heptad sequences (positions a and d in a sequence of a-g amino acids).^27^ A role of a disulfide bridge formed by the cysteins for the stabilization of the NFL dimer has not been described yet. Our data suggest such a role as reduction leads to release of monomers in denaturing conditions. In NFL, the single cysteine at aa 322 occupies a ‘d’ position in the heptad repeat sequence ‘a-g’ characteristic of coiled coils.^28^ The ‘d’ positions are pointed towards the inside of the coiled coil and are thus well positioned to form a disulfide bridge in a coiled coil NFL dimer. Moreover, the cysteine at position 322 may indeed have an important biological function for NFL, because sequence alignment of NFL shows that this feature is preserved among species (100% conservation among 9 species tested for various mammals and zebrafish). A more direct proof of the presence of a disulfide bridge stabilizing a dimer or a molecule bound to NFL would be the detection of enzymatic fragments containing this disulfide linkage. Work in this direction via search for peptides containing the -S-S- linkage is ongoing. Indirect evidence for the presence of a disulfide bridge in the immunoprecipitate was the detection of prominent adduct formation during reduction of the immunoprecipitate with DTT, which can react with disulfide bonds forming adducts but not with free thiol groups.^29^

Quite surprisingly, the Uman MAb UD3 which is the detector antibody of the Uman NFL ELISA kit^20^ did not detect the ∼8–12 kDa NFL species on the blot of reduced samples. Therefore, the epitope of this MAb must depend on an intact disulfide bridge of cysteine 322 as seen on the non-reduced samples (Fig. 6). Indeed, Shaw *et al.*^18^ mapped the epitopes of the Uman MAb ‘UD2’ and their MAb 1D44 also to the region near or including the cystein 322. However, in that paper, UD2 is referred to as the capture antibody MAb 47.3, which is a different MAb than UD3 (UD3 is biotinylated MAb 2:1) used in our experiments. The dependence of binding of UD3 to NFL on a non-reduced cysteine disulfide bridge could have implications for analyzing clinical samples if they contain NFL of different redox status as reduced NFL could be non-detectable in assays involving UD3. The structure of NFL assemblies or fragments containing cysteine apparently depends on the redox state of the cysteine group—as for example biotinylated UD2 (=UD3) does not bind to reduced NFL forms. Whether the binding site of UD3 is a local structure that changes dependent on the redox state, or whether it is a more overall structure that is affected by reduction is still unclear. If reduction results in a local effect on binding, then only one or very few MAbs would be changing their binding affinity whereas overall structural changes may affect the binding of several MAbs. Using antibodies as probes for changes of binding upon reduction may be quite useful to monitor structural changes (conformational changes or quaternary structure changes).

The homogeneous ELISA assay (Fig. 2) can only suggest the presence of a multimeric NFL assembly, but it cannot distinguish between dimeric and, for example, tetrameric molecules. One could also propose that the size difference of ∼22 kDa between the NFL species seen in reducing versus non-reducing gels might be due to complex formation between an NFL dimer and another protein or protein fragment. If we assume that the approximate MW of the NFL monomeric fragments is about 8–10 kDa, then an NFL dimer would be about 16–20 kDa. The difference of about 10–14 kDa to the MW of about 30 kDa of the complex would be accounted for by the unknown partner in the complex with the NFL dimeric fragment. Based on the literature, the most likely partners of NFL in a complex would be neurofilament heavy, neurofilament medium or α-internexin.^1^ However, the analysis of immunoprecipitated NFL by mass spectrometry did not reveal any unique peptides originating from those proteins, and currently no other candidate binding partners in the complex have been identified. This could either mean that the CSF samples did not contain any such heterocomplexes with NFL, or that the immunoprecipitating antibodies 1B11 and 6H63 did not recognize such heteromeric structures. Most, if not all anti-NFL antibodies have been generated using purified antigens of parts or the whole NFL, but not of heteropolymers of NFL with other neurofilament proteins. Therefore, it may be that heteropolymers have a different structure to those of the homopolymers, making them unrecognizable to the current NFL antibodies. This opens the possibility that intermediate filament heteropolymers could be used as antigens to generate new antibodies which would then recognize the presence of such structures in CSF. Likewise, end-specific MAbs recognizing the NFL fragments ending at aa 406 could be new tools to detect the presence of calpain-cleaved NFL in patient samples. The presence of the potential calpain-1 cleavage site between aa 406 and 407 can give clues to the involvement of calpain-1 in the degradation of NFL.

A major limitation of this study is that it is mostly immuno-based. We can only see in these experiments what the antibodies can bind to. Therefore, it will be necessary to complement this study with others, applying antibody-independent techniques for detection, such as various mass spectrometry techniques. The strength of the Western blot study presented here is that the sizes of the fragment species can be assessed directly and in dependence of their redox status.

Of interest would be also the structure of NFL assemblies in serum and plasma, but these are much more difficult matrices to investigate (about 40× lower levels of NFL in serum,^30^ and higher background binding of other proteins in immunoassays).

The knowledge about the molecular forms of NFL in CSF will be of importance for the quantitation of NFL in different immunoassays if the aggregation status of a sample is different from that of the calibrator used. If for example the calibrator consists of monomeric NFL but the NFL in a CSF sample is oligomeric, then the capture of one oligomeric molecule in the CSF sample can give signal from several reporter molecules in a sandwich assay, whereas one captured monomeric calibrator molecule could only bind one reporter molecule. Quantitation relative to the calibrator becomes also difficult if the aggregation state varied among patient samples. Knowledge about the molecular forms of calibrator and samples may help to interpret quantitative results from seemingly similar ELISA assays. If the fragment pattern varies among diseases or disease stages, assays targeting particular fragments could also enable development of more selective assays for neurodegenerative diseases.

## Supplementary Material

fcaf129_Supplementary_Data

## Data Availability

The datasets used and/or analyzed during the current study are available from the corresponding author on reasonable request.
