# Supplementary material for: Novel insights into the molecular nature of neurofilament light polypeptide species in cerebrospinal fluid
Source: Brain Commun. 2025 Apr 2;7(2):fcaf129. doi: 10.1093/braincomms/fcaf129 (PMC12003950; doi:10.1093/braincomms/fcaf129)

Supplementary Materials

Digestion conditions

In-gel digestion:

Fractionation on SDS-PAGE: aliquots from a immunoprecipitation of CSF (mixture of magnetic beads coated with either MAb 1B11 or 6H63; Encor Biotechnology) were separated in reduced and non-reduced conditions by SDS-PAGE (10% Bis Tris gel; Bio-Rad #3450112) along size markers in nearby lanes. After separation, the gel was stained in SimplyBlue™ (Thermofisher, # LC6065) stain, then destained briefly in water until the MW size marker proteins were clearly visible. Guided by the position of the size markers, the two lanes containing the separated immunoprecipitates were cut into each 11 consecutive gel blocks representing each different MW regions. The gel pieces were added to separate Eppendorf tubes (0.5 mL) and then dehydrated by adding 200 µL acetonitrile, followed by short finger flips. The liquid phase was discarded and the dehydration repeated once more. The gel pieces (without liquid) were then stored at 4 ºC.

For reduction, the gel pieces from the reduced sample lane were incubated in 100 µL of reduction buffer (10 mM DTT in 25 mM ammonium bicarbonate) for 30 min at 56 ºC on a shaker (800 rpm). The supernatants were removed and discarded. The gel pieces were dehydrated twice (10-15 min) with 200 µL acetonitrile, and the supernatants discarded.

Carbamidomethylation was performed by adding 50 µL carbamidomethylation solution (55 mM iodoacetamide in 25 mM ammonium bicarbonate) to the reduced gel pieces and incubation for 20 min at room temperature in the dark on a shaker (800 rpm).

Supernatants were removed and discarded. The gel pieces were then washed with 100 µL 50 mM ammonium bicarbonate for 10 min at 800 rpm. The liquid was then removed and discarded. The gel pieces were then again dehydrated (twice) with 160 µL acetonitrile for 5 min at 800 rpm.

For gel pieces from the lane containing the non-reduced sample the same procedures were performed, except that DTT and iodoacetamide solutions were replaced by buffer (25 mM ammonium bicarbonate).

<u>Digestion</u> was performed by adding 30 µL digestion solution Lys-C (Promega # VA117A; 12.5 ng/µL in 50 mM ammonium bicarbonate) to each of the dehydrated gel block samples on ice. After 30-45 min, 40 µL 25 mM ammonium bicarbonate was added to cover the gel pieces. The digestion was carried out by incubation of the gel pieces/Lys-C solution in closed Eppendorf tubes overnight at 37 ^{o}C.

<u>Extraction</u> of peptides from the gel digests: extraction 1: 60 µL 25 mM ammonium bicarbonate solution was added and the tubes and shaken for 10 min, 800 rpm, at room temperature. The supernatants were then transferred to separate 0.5 mL Eppendorf tubes. Extraction 2: 60 µL acetonitrile was added to the gel pieces and the tubes incubated for 15 min under shaking at 37 ^{o}C. After 5 min bench rest, the condensation droplets on the lids were spun down. The supernatants were transferred and combined with the initial supernatants. Extraction 3: 80 µL 5% formic acid was added to the gel pieces and incubated for 15 min under shaking at 37 ^{o}C. After another 5 min bench rest, the condensation droplets on the lids were spun down and the supernatants transferred and combined with the initial supernatants. Extraction 4: 60 µL acetonitrile was added to the gel pieces and incubated for 15 min under shaking at 37 ^{o}C. After 5 min bench rest,

the condensation was spun down and the supernatants combined with the initial supernatants. All collected extracts were then concentrated to dryness in a Savant Speedvac™ concentrator (Thermo Scientific).

Prior to MS analysis, the dried samples were dissolved in 50 µL sample buffer (0.05% trifluoro acetic acid, 0.1% BSA) by shaking for 10 min.

In-vitro **digestion of bovine NFL by calpain-1**

Bovine NFL (3.7 ng) was digested in a volume of 40 µL in 50 mM Tris (pH 7.5), 100 µM calcium chloride and various dilutions of calpain-1 (Millipore 208713) for 90 min at room temperature. The highest amount of calpain-1 added was 168 ng (= 1 µL of a 10-fold dilution of stock solution), then lowered in 3-fold steps. For negative control, calpain-1 was omitted. The reaction was stopped by the addition of 1 µL 50 mM EDTA. For mass spectrometric analysis, half of the digestion mixes (=20.5 µL) were further digested with each 20 ng Lys-C (Promega VA117A) over night at 37 °C. 10 µL of each digestion mix was then directly injected for mass spectrometry.

NFL sequence coverage

Supplementary Table 1, Supplementary Figure 1 and Supplementary Figure 2

Supplementary Table 1: **Sequence coverage of NFL** by peptides identified by mass spectrometry in Lys-C *in-vitro* digests of NFL immunoprecipitates from a pool of CSF. An aliquot of the immunoprecipitate has been reduced and carbamidomethylated before the digest ("red. + carbamido."), whereas the other aliquot has been directly digested ("non-red."). The latter aliquot has therefore not been reduced nor carbamidomethylated prior to digestion. Cystein-containing peptides are highlighted in yellow. The figure below shows the NFL sequence covered by the peptides identified.

Peptide table

Peptide	Area Sample 1 (red. + carbamido.)	Area Sample 2 (non-red.)	Start	End	PTM
VLEAELLVLRQK	3.27E+05	3.62E+05	117	128	
GADEAALARAELEK	5.84E+05	7.49E+05	198	211	
RIDSLMDEISFLK	9.76E+03		212	224	
DIRAQYEK		4.20E+04	260	267	
SRFTVLTESAAK	1.65E+06	1.94E+06	282	293	
FTVLTESAAK		4.53E+04	284	293	
TVLTESAAK	1.42E+05	1.35E+05	285	293	
NTDAVRAAKDEVSESRRLLK	2.38E+06	1.47E+06	294	313	
AVRAAKDEVSESRRLLK	3.53E+05		297	313	
AAKDEVSESRRLLK	2.36E+05	1.70E+05	300	313	

AKDEVSESRRLLK	1.34E+05	1.57E+05	301	313	
DEVSESRRLLK	5.82E+07	5.96E+07	303	313	
TLEIEAC(+209.02)RGMNEALEK	5.06E+06		316	331	Carbamidomethylated DTT
TLEIEAC(+57.02)RGMNEALEK	9.23E+05		316	331	Carbamidomethylation
TLEIEACRGMNEALEK		2.88E+04	316	331	
IEAC(+57.02)RGMNEALEK	1.69E+05		319	331	Carbamidomethylation
AC(+57.02)RGMNEALEK	3.49E+05		321	331	Carbamidomethylation
C(+57.02)RGMNEALEK	1.86E+05		322	331	Carbamidomethylation
QLQELEDK	7.05E+07	1.42E+07	332	339	
QNADISAMQDTINK	3.57E+07	1.99E+07	340	353	
ADISAMQDTINK	4.68E+04		342	353	
DISAMQDTINK	2.29E+04	2.52E+04	343	353	
AMQDTINK		6.70E+04	346	353	
LENELRTTK	8.04E+06	4.73E+06	354	362	
ENELRTTK	2.21E+05	1.07E+05	355	362	
NELRTTK	3.48E+04		356	362	
SEMARYLK	9.60E+06	5.56E+06	363	370	
MARYLK	7.13E+05	3.62E+05	365	370	
EYQDLLNVK	8.11E+07	7.65E+07	371	379	
EYQDLLNV	2.20E+04		371	378	
MALDIEIAAYRK	1.85E+06	5.93E+04	380	391	
LLEGEETRLS	3.61E+06	2.60E+06	392	401	
LLEGEETRL	8.48E+05	7.87E+05	392	400	
LLEGEETRLSFTSVG	8.67E+05		392	406	
LLEGEETRLSFT	8.75E+04		392	403	

Supplementary Figure 1: **NFL sequence covered by the Lys-C peptides identified.** Each of the blue bars represents a peptide identified by mass spectrometry (see also Supplementary Table 1).

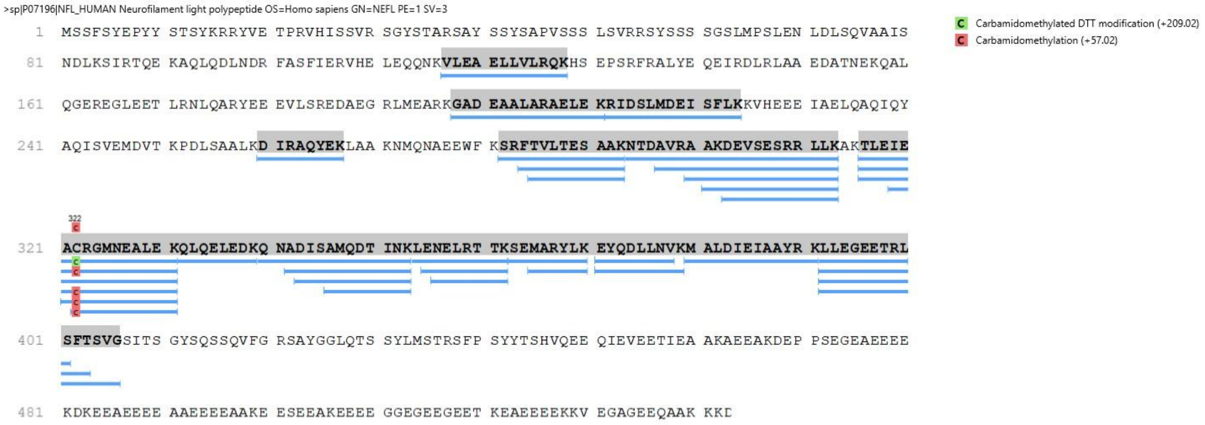

Supplementary Figure 2: Abundance of identified cysteine-containing peptides, with and without reduction and carbamidomethylation.

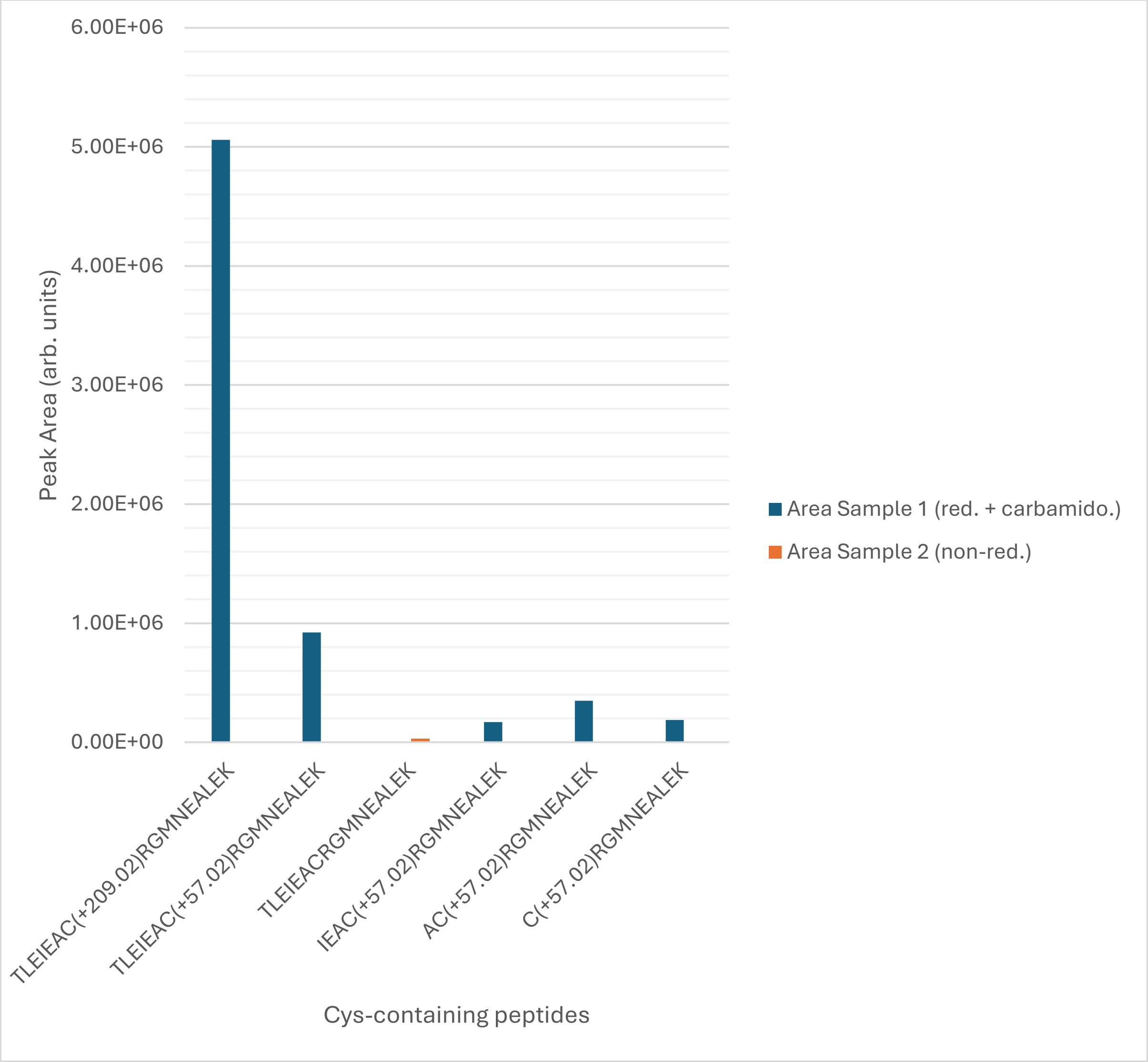

Supplementary Table 2: **Summary of the peptides identified in an *in-vitro* calpain-1/Lys-C digest of bovine NFL.** Peptides derived by both calpain-1 and Lys-C cleavage are listed in red, whereas Lys-C derived peptides are shown in black text. The peptide showing the presence of a calpain-1 cleavage site between aa 407/408 (corresponding to aa 406/407 in the human NFL sequence) is highlighted in yellow. The list contains additional calpain-1/Lys-C peptides (red) indicating the presence of multiple cleavage sites for calpain on the full-length bovine NFL preparation.

Checked	Confidence	Annotated Sequence (bovine NFL)	Master Protein Accessions	Positions in Master Proteins	Abundance calp dil 10	Abundance calp dil 30	Abundance calp dil 90	Abundance calp dil 270	Abundance no calp	Confidence (by Search Engine): Sequest HT	XCorr (by Search Engine): Sequest HT	Top Apex RT [min]
TRUE	High	[K].VHEEEIAELQAQIQYAQISVEMDVSSKPDLSAALK.[D]	P02548	P02548 [226-260]	193106	732042	1522247	1862047	203728	High	7.25	74.47
TRUE	High	[Y].TSHVQEEQIEVEETIEAAK.[A]	P02548	P02548 [445-463]		500616	477380	421200	56880	High	6.39	50.17
TRUE	High	[K].GADEAALARAELEK.[R]	P02548	P02548 [198-211]	3433962	2367938	1802940	2144458	1860089	High	5.29	39.36
TRUE	High	[K].AQLQDLNDRFASFIERVHELEQQNK.[V]	P02548	P02548 [92-116]			2445595	9924813		High	5.26	69.19
TRUE	High	[K].KVHEEEIAELQAQIQYAQISVEMDVSSKPDLS.[A]	P02548	P02548 [225-256]			100977	368148		High	4.53	70.78
TRUE	High	[K].VLEAELLVLRQK.[H]	P02548	P02548 [117-128]			381530	581564		High	4.52	54.67
TRUE	High	[K].RIDSLMDEIAFLK.[K]	P02548	P02548 [212-224]	1513527	1306371	1422847	1192867	1301984	High	4.5	69.72
TRUE	High	[K].LLEGEETRLSFTSVG.[S]	P02548	P02548 [393-407]	1393484	765119	401232	279802		High	4.47	58.95
TRUE	High	[G].SLMPSLESLDLSQVAAISNDLK.[S]	P02548	P02548 [63-84]				680257		High	3.13	82.13
TRUE	High	[K].LLEGEETRLSFTSVGSLT.[T]	P02548	P02548 [393-410]		763718	1272884	2311782		High	3.03	66.12
TRUE	High	[K].VHEEEIAELQA.[Q]	P02548	P02548 [226-236]		1094604	551936	339518	246724	High	2.79	43.61
TRUE	High	[K].DEPPSEGEAEEEK.[E]	P02548	P02548 [469-482]	330278	178826	326397	310785	29097	High	2.71	31.62
TRUE	High	[K].SEMARYLK.[E]	P02548	P02548 [364-371]	569119	361222	599851		235893	High	2.7	31.27
TRUE	High	[K].SRFTVLTE.[S]	P02548	P02548 [283-290]	1914767	1248543				High	2.46	43.54
TRUE	High	[RKQ].EYQDLLNVK.[ML]	O77788; P02548	O77788 [383-391]; P02548 [372-380]	1848248	1735383	1954032	1774227	1564045	High	2.34	49.07
TRUE	High	[R].SGYSTARSAYSSY.[S]	P02548	P02548 [31-43]	806900	749316	436732	168929		High	2.34	34.89
TRUE	High	[K].LENELRTTK.[S]	P02548	P02548 [355-363]	506781	491413	485615	530135	471699	High	2.1	26.42

Supplementary Figure 3. Uncropped version of the Western blot showed in Figure 3.

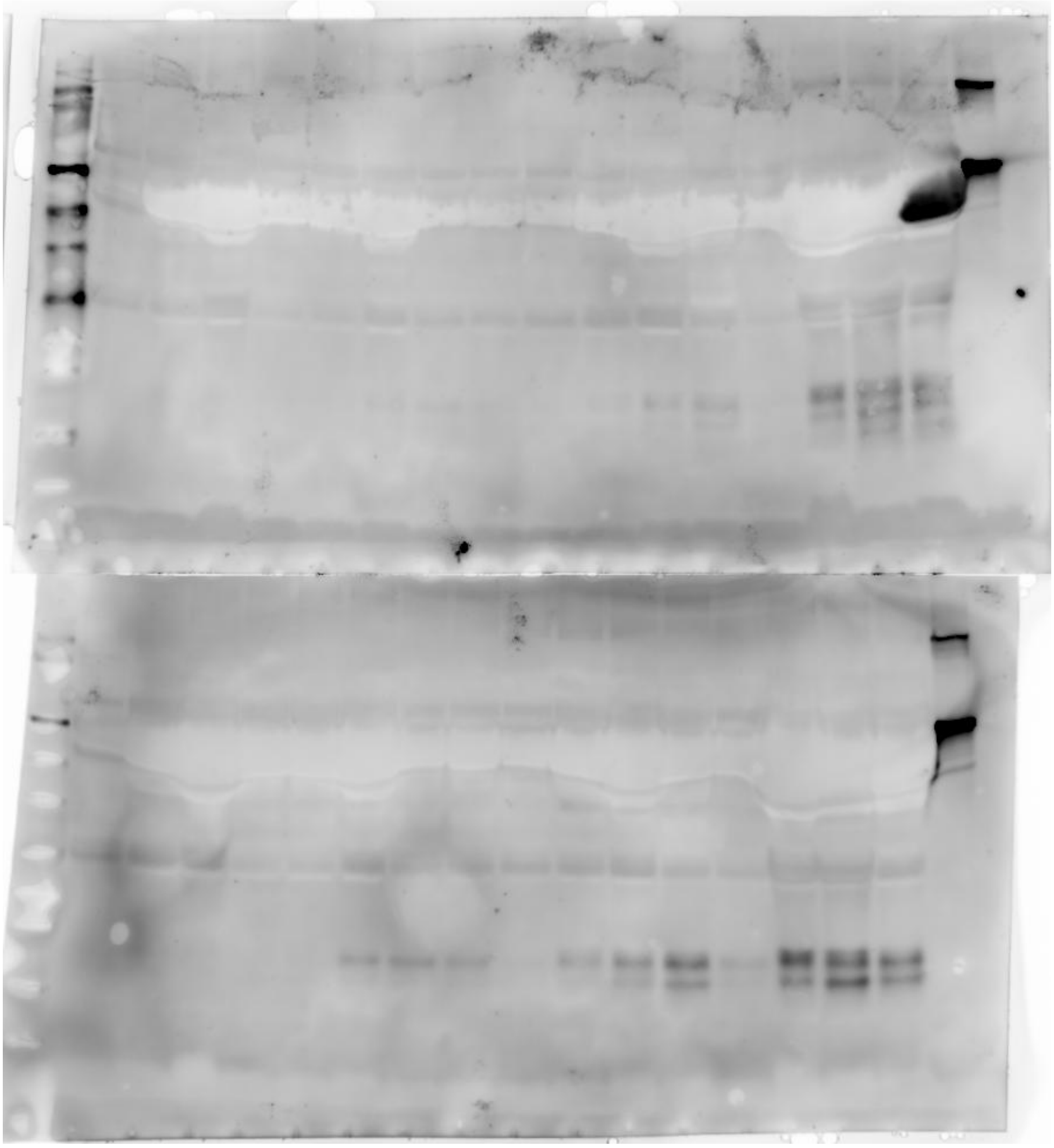

Supplementary Figure 4. Uncropped version of the Western blot showed in Figure 4.

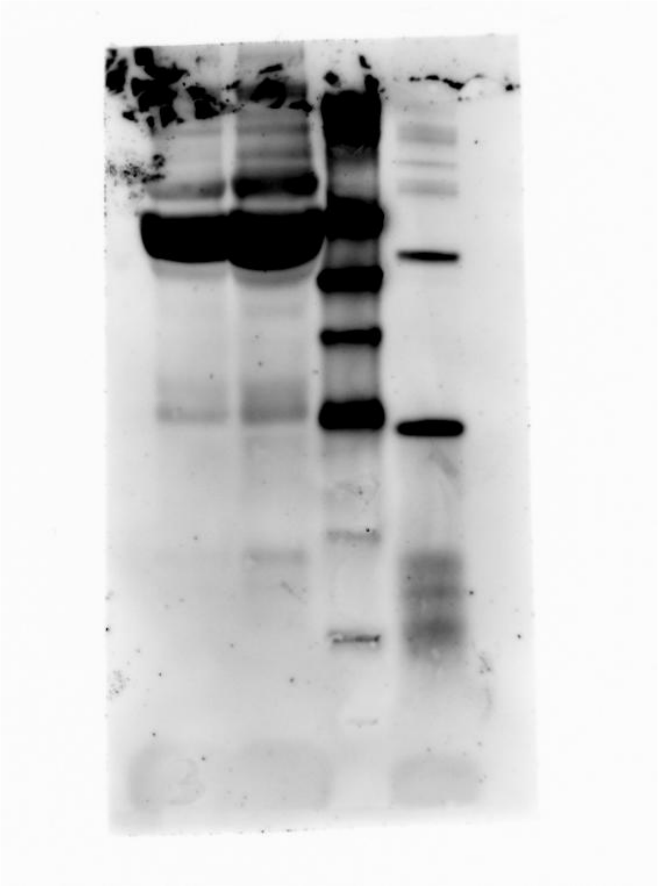

Supplementary Figure 5. Uncropped version of the Western blot showed in Figure 5.

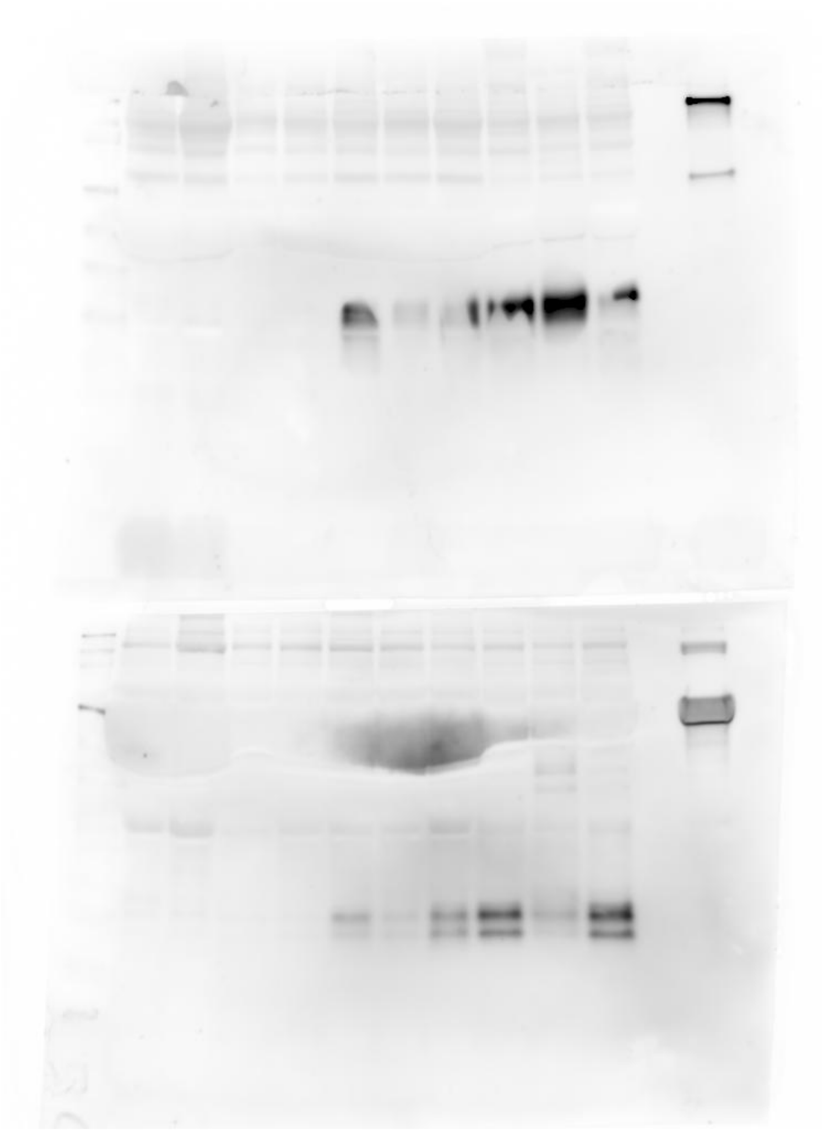

Supplementary Figure 6. Uncropped version of the Western blot showed in Figure 6.

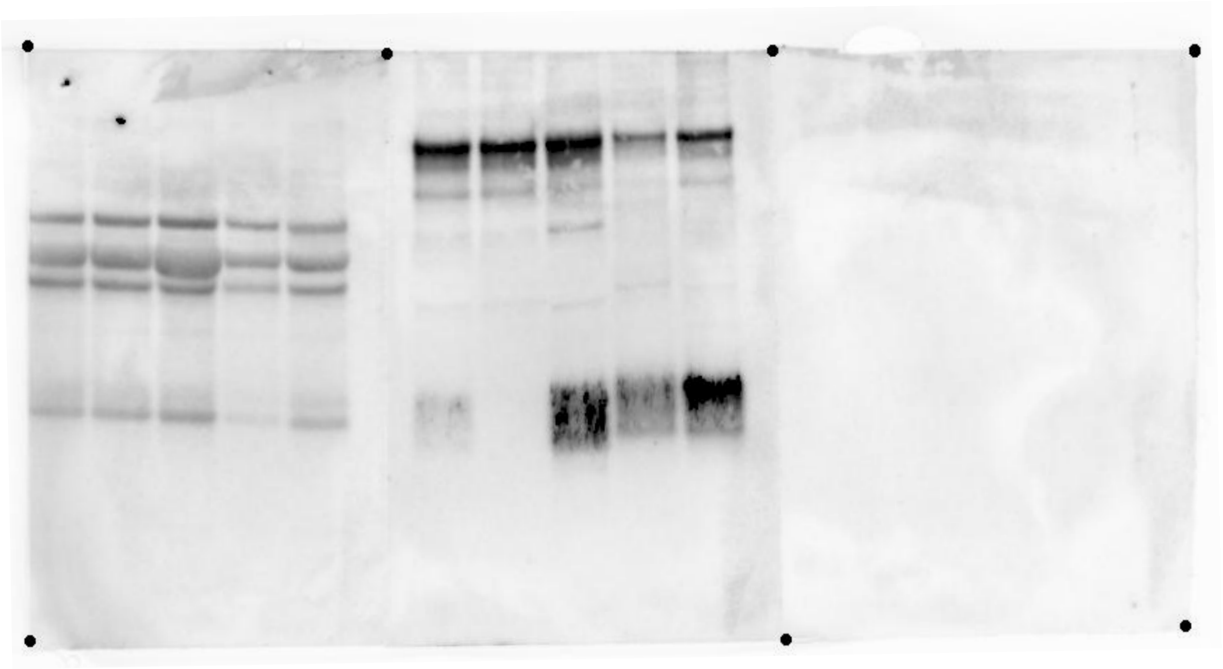

Supplement: fcaf129_Supplementary_Data [file fcaf129_supplementary_data.pdf]
